# Synthesis, crystal structure, and anti-breast cancer activity of a novel metal-porphyrinic complex [YK(TCPP)(OH)_2_·(solvents)_x_]

**DOI:** 10.1590/1414-431X20176858

**Published:** 2017-11-13

**Authors:** J-C. Liu, J-J. Gu, Y-S. Zhang

**Affiliations:** 1 Department of Radiotherapy, Huaihe Hospital of Henan University, Kaifeng, Henan, China; 2 Department of Oncology, Huaihe Hospital of Henan University, Kaifeng, Henan, China

**Keywords:** Heterometallic, Single crystal, Breast cancer cell

## Abstract

A novel heterometallic metal-porphyrinic framework (MPFs) built from Y and K ions as nods and *meso-*tetra(4-carboxyphenyl)porphyrin as linkers has been successfully synthesized and characterized. The single crystal X-ray diffraction indicated that this complex 1 exhibited a bilayered architecture of the porphyrins, which is seldom seen in MPFs. In addition, *in vitro* anticancer activity of complex 1 on three human breast cancer cells (BT474, SKBr-3 and ZR-75-30) was further determined.

## Introduction

Metal-organic frameworks (MOFs) are a class of porous crystalline materials that are constructed from judiciously designed multi-dentate organic linkers and discrete metal ions/clusters (1,2). Due to their structural diversity and tunability as well as their promising applications in gas storage/separation (3,4), chemical sensing (5), heterogeneous catalysis (6,7), and biomedical usage (8), etc., MOFs have attracted tremendous research interest in the past two decades. Among the vast family of organic ligands used in constructing MOFs, porphyrins are a family of biologically relevant tetrapyrrole macrocycles (9,10). Using porphyrins as linkers to build crystalline multidimensional assemblies via coordination bonds gives rise to a sub-branch of MOFs, the metal-porphyrinic frameworks (MPFs) (11,12). Since the first report in 1991 by Robson and co-workers (13), MPFs have attracted increasing research interest due to their intriguing properties. Fabricating crystalline MPFs from porphyrins could introduce the unique electronic, photochemical and catalytic properties of porphyrins to the resulting porous material, thus making MPFs multi-functional. For example, MPFs have demonstrated great potential for applications in artificial light-harvesting systems (14), pH sensing (15), photodynamic therapy (16), heterogeneous catalysis (17), etc. However, despite the research attention to synthesize novel MPFs and develop their underlying applications, the development of MPFs is still in its beginning (18). The number of MPFs is still scarce and the construction of novel MPFs with diverse structures is highly desirable (19 [Bibr B20]).

**Figure 1.**
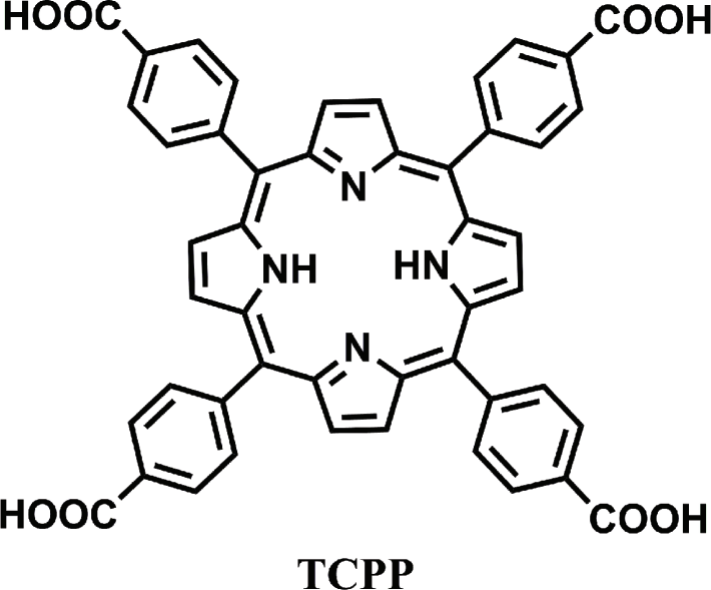
*Meso*-(4-carboxyphenyl)porphyrin (TCPP) used in this research.

In this study, we report the synthesis and structure of a novel heterometallic MPF 1. Complex 1 was constructed from a frequently used porphyrin ligand *meso-*tetra(4-carboxyphenyl)porphyrin (TCPP, Figure 1), yttrium and potassium ions. X-ray single crystal diffraction revealed that complex 1 possessed a bilayered arrangement of the porphyrins, which is rarely seen in MPFs. In addition, the anticancer activity of complex 1 was then evaluated on three human breast cancer cells (BT474, SKBr-3 and ZR-75-30) in an *in vitro* experiment.

## Material and Methods

### Apparatus and materials

All starting materials and reagents used in this work were obtained commercially and used without further purification. Element analyses (C, H, and N) were determined with an elemental Vairo EL III analyzer (Bruker, Germany). The powder X-ray diffraction patterns (PXRD) of complex 1 were recorded on a Rigaku D/Max-2500 diffractometer (Bruker), operated at 40 kV and 100 mA, using a Cu-target tube and a graphite monochromator. The intensity data were recorded by continuous scan in a 2θ/θ mode from 5° to 50° with a step size of 0.02° and a scan speed of 8°/min. Simulation of the PXRD spectra was carried out by the single-crystal data and diffraction-crystal module of the Mercury (Hg) program available free of charge via the Internet at http://www.iucr.org. Single crystal X-ray diffraction was carried out by an Oxford Xcalibur E diffractometer. Three human breast cancer cells (BT474, SKBr-3 and ZR-75-30) were purchased from the National Institute for the Control of Pharmaceutical and Biological Products (China).

### Synthesis of [YK(TCPP)(OH)_2_·(solvents)_x_] complex 1

TCPP (20 mg, 0.024 mmol) was added into N,N-diethylformamide (4 mL) in a small capped vial and sonicated for 10 min for dissolution. A 0.5 mL deionized water and 10 mg of Y(NO_3_)_3_·6H_2_O (0.026 mmol) were added into the above solution and further sonicated for 10 min. Then three drops of 1 M KOH solution was added into the above mixture to adjust the pH value. The vial was placed into a Teflon-lined acid digestion bomb and heated at 120°C for 3 days, and then it was allowed to cool to room temperature naturally. Small rodlike crystals of complex 1 were obtained followed by washing several times with ethanol and anhydrous ether, respectively. Yield: ∼10.5 mg (46%, based on porphyrin). Elemental analysis (%) found (calcd) for complex 1 (C_96_H_56_K_2_N_8_O_20_Y_2_): C, 60.58 (60.77); H, 2.91 (2.97); N, 555 (5.91).

### Crystal structure determination

According to the evaporation of chloroform solution, suitable single crystals of complex 1 were formed. The diffraction data were acquired on a Bruker Smart Apex CCD area detector using a graphite monochromated Mo Kα radiation (*λ* = 0.71073 Å) at room temperature. The structure was solved by using the program SHELXL-97 (14) and Fourier difference techniques, and refined by full-matrix least-squares method on *F* ^2^. Hydrogen atoms were placed in appropriate positions using a riding model. Crystallographic data for complex 1 is shown in Table 1.

**Table 1.** Crystal data, data collection and structure refinement of complex 1.

Formula	C_96_H_56_K_2_N_8_O_20_Y_2_
*M*r	1897.50
Crystal system	Monoclinic
Space group	*P*2/*m*
*a*/Å	11.821 (2)
*b*/Å	17.139 (3)
*c*/Å	16.480 (2)
*α*/°	90
*β*/°	108.008 (3)
*γ*/°	90
*V*/Å^3^	3175.2 (9)
*Z*	1
*D*_calc_/g·cm^-3^	0.992
*μ*(Mo Kα)/mm^-1^	1.028
*θ* range/°	2.167 to 24.999
Reflections collected	21800
No. unique data [*R*(int)]	5762 [0.0743]
No. data with *I* ≥ 2*σ*(*I*)	4001
*R*_1_	0.1260
*ωR*_2_(all data)	0.3657
CCDC	1555392

### Antitumor activity

Three human breast cancer cells (BT474, SKBr-3 and ZR-75-30) and one normal cell line (MDCK) were determined using the MTT assay. In this study, the cells were plated on 96-wells at 5×10^3^. After attachment (24 h), the cells reaching 70–80% confluency were treated for 48 h with each compound at different concentrations or 1% dimethyl sulfoxide (DMSO) as the negative control. After 48 h incubation, 20 μL of MTT solution (5 mg/mL in PBS) was added and incubated for an additional 4 h. Subsequently, the medium was aspirated carefully, and 150 μL of DMSO was added. After incubation for 15 min, absorbance was measured at 490 nm using FlexStation 3 benchtop multi-mode microplate reader (Molecular Devices, USA). This assay measures the amount of formazan produced from MTT by the dehydrogenase enzymes of metabolically active cells. Thus, the quantity of formazan produced is directly proportional to the number of living cells. Absorbance values of the treated cells were compared with the values of untreated cells. The IC_50_ value was determined from non-linear regression equation. The results are presented as the average percentage viability to the negative control (1% DMSO).

## Results and Discussion

### Molecular structure

**Figure 2.**
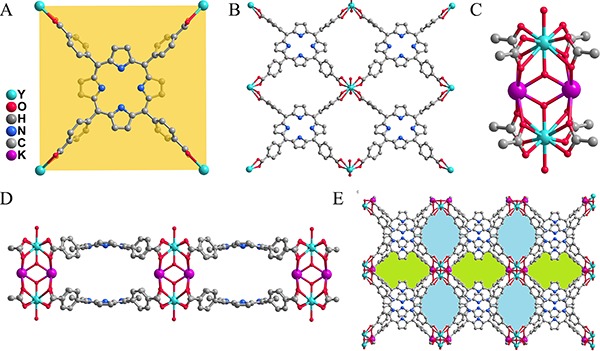
*A*, Coordination environment of TCPP ligand in complex 1, the linker exhibit a saddled out of plane deformation. The yellow plane represents the mean porphyrin plane created through pyrrolic N atoms. *B*, The infinite 2D sheet formed by the connection of Y ions and TCPP linker. *C*, Coordination environments of Y and K ions. *D*, Side view of the double-sheeted layer formed by the connection of K ions and porphyrin sheet, illustrating the bilayered arrangement of the porphyrins. *E*, Channels propagate along the *a*-axis, green color: 5.4×13.4 Å, blue color: 13.3×8.7 Å, atom to atom distance.

Dark purple rod-like single crystals of complex 1 were obtained *via* solvothermal reactions. Single-crystal X-ray diffraction studies disclosed that the complex crystallized in the monoclinic *P*2/*m* space group. As depicted in Figure 2A, the benzoate arms of quadrangular porphyrin ligand TCPP were fully deprotonated to coordinate to four Y ions, making TCPP to serve as a tetra-dentate linker. The phenyl rings in each side arm of the porphyrin linker are tilted to the porphyrin plane by 64.66°, 65.13°, 56.63°, and 69.38°. The porphyrinic core of TCPP was free without *in-situ* metallization during the solvothermal process. An obvious phenomenon was that the TCPP linker exhibited a saddled type out-of-plane distortion with a maximum deviation of ∼0.495 Å, which means that the β-carbons on each pyrrolic ring are alternatively above and below the porphyrin mean plane while the pyrrole nitrogens are on the plane. Meanwhile, each Y^3+^ ion was coordinated to four carboxylate groups from different porphyrin ligands and one water molecule as well as one μ_3_-OH group, resulting in a 10-connected structural nod. The connection of TCPP linkers and Y^3+^ nodes originated an infinite 2D sheet that propagated along the *b*-axis (Figure 2B). Interestingly, K^+^ ions, which came from KOH solution in the synthesis process, were also observed in complex 1. Each K^+^ is coordinated to four oxygen atoms from adjacent carboxylate groups of TCPP and two oxygen atoms from neighboring μ_3_-OH groups, resulting in an octahedral geometry (Figure 2C). As shown in Figure 2D, because of the connection effect of K^+^ ions, every adjacent 2D porphyrin sheet was linked together to form a double-sheeted geometry with inter-sheet channel voids of 6.1×17.1 Å (atom to atom distance). However, these double-sheeted layers were not inter-connected with each other and the adjacent layer gave an …AA… packing sequence with the interlayer distance of 3.15 Å. As shown in Figure 2E, the packing of these bilayered porphyrin aggregates also gives rise to large rhombic channels of 5.4×13.4 and 13.3×8.7 Å in diagonal dimensions propagating along the *a*-axis. Thus, the resulting architecture is characterized by inter-porphyrin channels, which extend not only perpendicular to the porphyrin frameworks, but also parallel to them within the bilayered aggregates. The solvent accessible pore volume comprises 43.7% of its unit cell volume as calculated by Platon. The overall double-layered geometry of complex 1 was similar to a previously reported structure that was constructed from TCPP linker and sodium ion, which represents a seldom seen architecture in MPFs (7,8). Moreover, there are the N-H…N and C-H…O hydrogen bonds in the packing structure. The detailed information of hydrogen bonds is reported in Table 2.

**Table 2.** Hydrogen bonds information (Angstrom, Deg).

D–H…A	D–H	H…A	D…A	D–H…A	Symmetry code
N2–H2…N1	0.8800	2.3600	2.893(10)	119.00	
N2–H2…N1	0.8800	2.3600	2.893(10)	119.00	x, 2-y, z
N3–H3…N1	0.8800	2.3600	2.898(10)	120.00	
N3–H3…N1	0.8800	2.3600	2.898(10)	120.00	x, 2-y, z
C3–H3A…O3	0.9500	2.5000	2.84(3)	101.00	
C3–H3A…O3	0.9500	2.3900	3.33(3)	170.00	1-x, y, -z
C18–H18…O7	0.9500	2.4400	2.79(2)	101.00	

**Figure 3.**
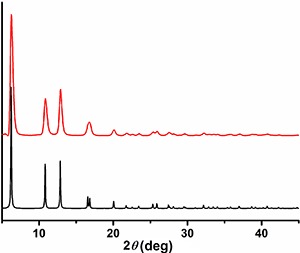
Powder X-ray diffraction patterns for complex 1 (above: simulated curve; below: experimental curve).

To investigate whether the analyzed crystal structures are truly representative of the bulk materials, PXRD was performed for complex 1 at room temperature (Figure 3). The main peak positions observed were in good agreement with the simulated ones. Although minor differences could be found in the positions, widths, and intensities of some peaks, it could still be considered that the bulk synthesized materials and the analyzed crystal were homogeneous. The differences may be due to the preferred orientation of the powder samples.

### Anticancer activity

The cell growth inhibitory activity of the title complex 1 and organic ligand TCPP was evaluated against three human breast cancer cells (BT474, SKBr-3 and ZR-75-30) using standard MTT assay *in vitro*, with cisplatin as the positive control. Additionally, cytotoxic assays were conducted using normal cell line MDCK. The mean values of experiments from three independent determinations, reported as half-maximal inhibitory concentration (IC_50_) values, are summarized in Table 3. Complex 1 showed good antitumor activities (IC_50_=20∼30 μM), which was similar to that of cisplatin on the three cell lines, while its organic ligand proved to be ineffective against all three cell lines (IC_50_>100 μM). In addition, it is worth noting that complex 1 presented no inhibitory activity against MDCK cell lines (IC_50_>100 μM), which suggests that the compound exhibited good selectivity towards cancer cells rather than normal cells.

**Table 3.** Growth inhibitory effects of complex 1 and TCPP on BT474, SKBr-3, ZR-75-30 and MDCK cell lines.

Compounds	IC_50_±SD (μM)			
	BT474	SKBr-3	ZR-75-30	MDCK
TCPP	>100	>100	>100	>100
Complex 1	20.2±1.6	25.9±1.9	30.6±2.3	>100
Cisplatin	23.3±2.2	24.1±1.7	29.9±2.1	>100

Data are reported as means±SD of three independent determinations. Cisplatin was the positive control.

We concluded that, compared with the organic ligand TCPP, the anticancer activity of complex 1 was much improved, which might be ascribed to the coordination of TCCP ligand with the Y ion. The present study contributes to further design and synthesize Y(III) complexes that show potent antitumor effects.
